# TEGAFIRI is an effective alternative regimen for the management of recurrent or metastatic colorectal cancer

**DOI:** 10.3892/ol.2015.2855

**Published:** 2015-01-07

**Authors:** TZU-CHI HSU

**Affiliations:** Department of Surgery, Mackay Memorial Hospital, Zhongshan, Taipei 104, Taiwan, R.O.C.

**Keywords:** uracil-tegafur, irinotecan, metastatic, colorectal cancer

## Abstract

At present, the global incidence of colorectal cancer is increasing, with numerous individuals succumbing to the disease. The standard treatment strategy for colorectal cancer is curative resection. However, a cure is rarely achieved for metastatic colorectal cancer. Currently, chemotherapy is the main treatment for metastatic and recurrent colorectal cancer. The majority of metastases or recurrences have been found to respond well to chemotherapy. The present study evaluated the response rates of recurrent or metastatic colorectal cancer patients treated with a combination chemotherapy of irinotecan and oral uracil-tegafur (UFUR). In the pilot study, 33 patients with metastatic or recurrent colorectal cancer were treated with different regimens of irinotecan and UFUR with or without leucovorin; however, irinotecan (150 mg/m2 every two weeks) with continuous UFUR and leucovorin without interruption resulted in improved survival compared with the other regimens evaluated and, thus, was employed for the present study of 113 patients. The patients that received irinotecan with UFUR and leucovorin without interruption exhibited similar efficacy in terms of overall survival and response rate to that of the pilot study. In addition, the incidences of diarrhea, alopecia and hematologic toxicity were acceptable, which was in agreement with the results of the pilot study. Therefore, combination chemotherapy with irinotecan, oral UFUR and leucovorin appears to be a satisfactory treatment strategy for recurrent or metastatic colorectal cancer.

## Introduction

Currently, the preferred treatment strategy for metastatic and recurrent cancer is chemotherapy (1–4), as numerous types of metastatic and recurrent cancer appear to respond well to this mode of therapy (1–6). In addition, multidrug therapies result in improved responses compared with single agent therapies.

The aim of the present study was to evaluate the employment of irinotecan and oral uracil-tegafur (UFUR) (TEGAFIRI) with leucovorin to treat metastatic and recurrent colorectal cancer. To decrease bias, all of the patients in the present study were analyzed by a single health worker. As previous case studies reported variation in the dosage of irinotecan and the dosing schedule oral UFUR with or without leucovorin (10–14), a pilot study of recurrent or metastatic colorectal cancer patients was conducted by Hsu (15) to evaluate the response rates of different combination chemotherapy regimens of TEGFIRI with or without leucovorin, and to determine the optimal regimen dose. Thus, the present study employed the optimal regimen dose in a larger patient population to clarify the results of the pilot study.

## Materials and methods

### Treatment regimen

In the present study, 113 metastatic or recurrent colorectal cancer patients from Mackay Memorial Hospital (Taipei, Taiwan) were treated with a combination of irinotecan (Pfizer, Inc., New York, NY, USA) and UFUR (TTY BioPharm, Co., Ltd., Taipei, Taiwan) with or without leucovorin. Each UFUR capsule contained a 1:4 molar ratio of the 5-fluorouracil (5-FU) prodrug tegafur (100 mg) and the dihydropyrimidine dehydrogenase inhibitor uracil (224 mg). Each leucovorin tablet (TTY BioPharm, Co., Ltd.) contained 15 mg leucovorin. The present study was approved by the Institutional Review Board of the National Health Bureau of Taiwan (Taipei, Taiwan) and all of the patients provided written informed consent prior to receiving chemotherapy treatment.

### Patient selection

The patients selected to participate in the present study were aged ≥18 years and exhibited histologically determined colorectal cancer, characterized by a minimum of one measurable lesion and an Eastern Cooperative Oncology Group performance status of 0 or 1 (16). Prior to study enrollment, the following inclusion criteria were determined: A Karnofsky performance status of ≥80% (17), ≤2.0 mg/dl bilirubin, ≤1.5 mg/dl creatinine, an absolute granulocyte count of ≥1500/μl, and a platelet count of ≥100,000/μl. Patients who had not received chemotherapy in the six months prior to the present study were included; however, patients who had undergone chemotherapy for metastatic colorectal cancer, who exhibited central nervous system metastasis or had a life expectancy of less than three months were excluded. All 113 patients were monitored from January 2006 until December 2010 or mortality.

Patients eligible for the pilot study (15) received different regimens and doses of TEGAFIRI with or without leucovorin. The patients were randomized into three groups: Group I, 150 mg/m^2^ irinotecan every two weeks and UFUR for one week every two weeks; group II, 100 mg/m^2^ irinotecan for two weeks followed by a one week of rest, with continuous UFUR and leucovorin; and group III, 150 mg/m^2^ irinotecan every two weeks with continuous UFUR and leucovorin. The UFUR dose was standardized at 300 mg/m^2^/day and leucovorin was administered at 45–60 mg/m^2^/day. Although the intention was to include a greater number of patients in the pilot study, the initial results demonstrated that group I patients exhibited lower response rates compared with the other two groups; therefore, it was unethical to proceed with the group I regimen and the enrollment was terminated after 33 patients had enrolled. From the pilot study it was determined that that the group III regimen was optimal for the treatment of metastatic or recurrent colorectal cancer; thus, this regimen was administered to patients eligible for the present study.

In the present study, all of the 113 enrolled patients were treated with irinotecan at a standard dose of 150 mg/m^2^ every two weeks with continuous UFUR (300 mg/m^2^/day) and leucovorin (45–60 mg/m^2^/day). The Dukes’ stage of colorectal cancer before recurrence was determined and evaluated for each patient (18). In addition, prior to irinotecan administration, patients were administered with 10 mg dexamethasone intravenously, 3 mg granisetron or 8 mg ondansetron intravenously, and 0.5 mg atropine subcutaneously. Supportive care included loperamide for diarrhea, antiemetic agents and oral cephradine for diarrhea lasting >48 h. Upon progression, patients were administered with an oxaliplatin-based salvage regimen in addition to UFUR and leucovorin (TEGAFOX); however, agents such as bevacizumub and cetaximub were not routinely used due to a lack of funding during the study period.

### Patient monitoring and follow-up

In the pilot study, patients in each group were evaluated by determining the serum carcinoembryonic antigen (CEA) and performing a chest X-ray, abdominal ultrasound and computed tomography scan of the chest or abdomen every three months. In addition, the Response Evaluation Criteria In Solid Tumors (RECIST) was used to assess the efficacy of each chemotherapy regimen by categorizing the response into the following four grades: Progression, stable disease, partial response and complete response (19). Progressive disease was defined as an increase in CEA levels, a ≥25% increase in the number or size of the metastatic lesions or the development of new lesions; a partial response was defined as a decrease in CEA levels or a ≥25% decrease in the number or size of the metastatic lesions; and a complete response was defined CEA levels with the normal range or as the disappearance of metastatic lesions.

### Dose modification

The severity of the adverse effects was evaluated using the National Cancer Institute Toxicity Criteria (version 2.0) (20). Upon the initial appearance of grade II toxicity, no dose reduction was required. In addition, upon the appearance of reactions, which were determined as unlikely to become serious or life-threatening, no treatment interruption or dose reduction was implemented. However, in cases of grade III or greater toxicity, TEGAFIRI/leucovorin treatment was interrupted and was not resumed until the toxicity had resolved or had improved to grade I. When treatment was resumed, the dose of leucovorin was as before; however the doses of irinotecan and UFUR were reduced as follows: Irinotecan and UFUR doses were reduced by 25% in patients who exhibited a second occurrence of grade II toxicity or any occurrence of grade III toxicity; and irinotecan and UFUR were reduced by 50% in patients who experienced a third occurrence of grade II toxicity or a second occurrence of grade III toxicity. Treatment was discontinued if, despite dose reduction, grade II toxicity occurred for a fourth time, grade III toxicity occurred for a third time or if grade IV toxicity occurred at all. If granulocytes decreased to <500/mm^3^, grade III–IV diarrhea developed or granulocytes decreased to <1,000/mm^3^ with concomitant fever, irinotecan and UFUR doses were reduced by 20% for one cycle.

### Statistical methods

The baseline characteristics of the patients were quantified using descriptive statistics (median, percentile, and range) and the principle results were overall survival and progression-free survival. Overall survival was defined as the period of time from the commencement of irinotecan therapy to mortality. Progression-free survival was defined as the period of time from group randomization to disease progression, or mortality from disease progression or an unknown cause. For multivariate analysis, factors associated with the time to progression were identified by performing Cox’s regression analysis with forward stepwise conditional analysis. Furthermore, the progression-free and overall survival curves were calculated according to the Kaplan-Meier method and compared using a log-rank test. All statistical analyses were performed using SPSS software version 17.0.1 (SPSS, Inc., Chicago, IL, USA). P<0.05 was considered to indicate a statistically significant difference.

## Results

### Pilot study

**Figure 1:**
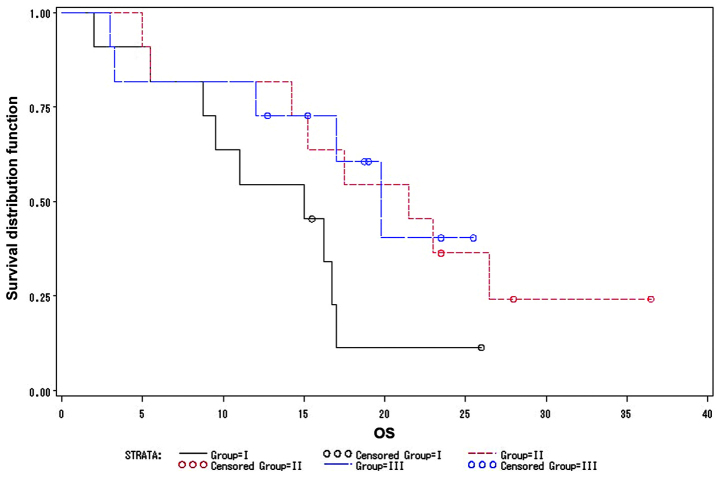
Overall survival curve of the three groups in the pilot study (P=0.1198 among the groups) (23).

In the pilot study (15), no significant differences were identified between the three groups in terms of dose intensity and dose delivery. Groups II and III exhibited improved response rates compared with group I (Table I). The overall survival times of groups I, II and III were 15, 21.5 and 19.8 months, respectively (Fig. 1). No statistically significant differences were identified between the three groups in terms of overall survival; however, group III patients exhibited fewer non-hematological side effects and improved tolerance to the regimen compared with groups I and II. Therefore, the group III regimen from the pilot study was selected for use in the present study (n=113).

### Present study

**Figure 2:**
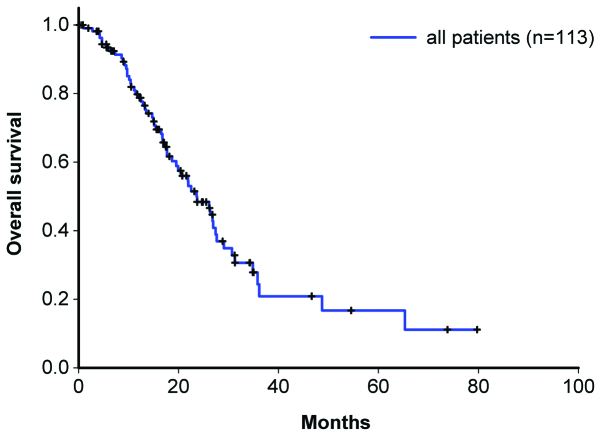
Overall survival curve of the patients in the present study.

**Figure 3:**
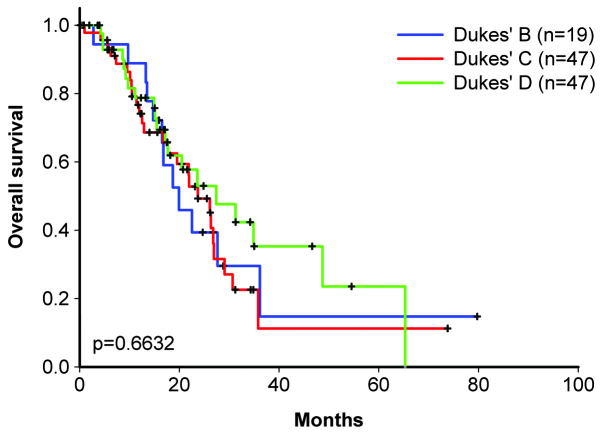
Overall survival curve of the patients in the present study by Dukes’ stage prior to recurrence. The overall survival was 19.9, 23.7 and 27.4 months for Dukes’ stages B, C and D, respectively.

**Figure 4:**
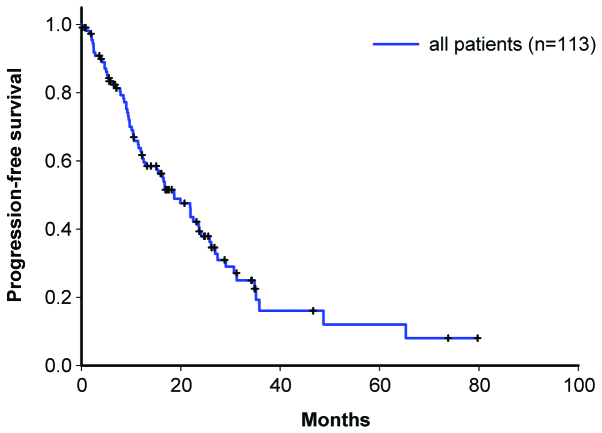
Progression-free survival curve of the patients in the present study.

**Figure 5:**
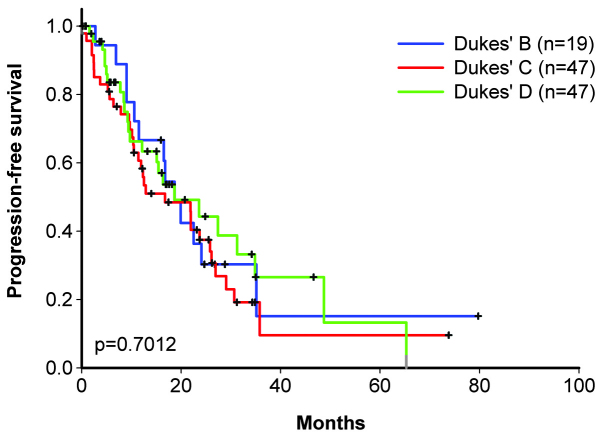
Progression-free survival curve of the patients in the present study by Dukes’ stage prior to recurrence. The progression-free survival was 18.7, 16.8 and 18.7 months for Dukes’ stages B, C and D, respectively.

Table II indicates the demographic data of the 113 patients enrolled in the present study. The ratio of patients with initial Dukes’ stage B:C:D was 19:47:47. According to the RECIST criteria, the response rate of the present study was 52.2%, which is similar to that of the pilot study (54.5%). In addition, the incidence of diarrhea, alopecia and hematologic toxicities (Table III), as well as the necessity of delaying or decreasing the dosage were similar to those of the pilot study. For Dukes’ stages B, C and D, the overall patient survival time was 19.9, 23.7 and 27.4 months, respectively (Figs. 2 and 3), and the progression-free survival time was 18.7, 16.8 and 18.7 months, respectively (Figs. 4 and 5).

## Discussion

As the worldwide incidence rate of colorectal cancer increases, increasing numbers of patients are succumbing to the disease (21,22). In 2012, there were approximately 694,000 mortalities as a result of colorectal cancer (23), and the American Cancer Society have estimated that 136,830 novel cases of colorectal cancer will be diagnosed and 50,310 mortalities as a result of the disease will occur in 2014 (24). In Taiwan, colorectal cancer is the third leading cause of cancer-related mortality and the second most common cause of malignancy. The principal treatment strategy for colorectal cancer is curative resection; however, remission of metastatic colorectal cancer is rarely achieved (25). Therefore, chemotherapy is currently employed as the preferred treatment strategy for metastatic disease (1–6).

A novel inhibitor of the DNA enzyme topoisomerase I, irinotecan exerts cytotoxic activity by interrupting DNA replication and transcription. In studies conducted in Western countries, TEGAFIRI with leucovorin administration resulted in response rates of 25–40% (4,6). Furthermore, good response or survival rates have been observed in a number of Taiwanese studies of first- and second-line irinotecan therapy. However, the addition of 5-FU or its precursors plus leucovorin were crucial for achieving satisfactory response rates.

Tegafur is an oral fluoropyrimidine, which is metabolized to 5-FU *in vivo* (26). In the management of metastatic colorectal cancer, tegafur appears to be an active and minimally toxic alternative to other types of fluoropyrimidine (27). Additionally, uracil is a naturally occurring pyrimidine, which is able to incorporate into nucleic acids (28). Together, these agents may be administered as oral UFUR, which consists of tegafur combined with uracil in 4:1 molar ratio. Preclinical studies have demonstrated that this combination of tegafur and uracil is associated with higher plasma levels of 5-FU compared with tegafur treatment alone (29,30). Furthermore, this difference in 5-FU plasma levels was associated with greater antitumor activity (29,30). Two phase III studies comparing UFUR/leucovorin with 5-FU/leucovorin demonstrated that the response rate, time to progression and overall survival time were similar between the two regimens, with an overall survival of 12–13 months. However, diarrhea, nausea and vomiting, stomatitis and mucositis, and myelosuppression occurred significantly less frequently in the UFUR/leucovorin compared with the 5-FU/leucovorin group (27,31).

In a number of Japanese and Taiwanese studies, UFUR was administered in favor of 5-FU as an agent in combination chemotherapy; for example, in the FOLFIRI regimen, UFUR replaced 5-FU in combination with irinotecan (9,32,33). In these previous studies, the dose of irinotecan (70, 80, 100, 150, 180, 220 or 350 mg/m^2^) and the interval between the doses (for example, once weekly, once every two weeks and once every three weeks) varied broadly.

Although leucovorin appears to enhance the antitumor efficacy of 5-FU in the treatment of metastatic colorectal cancer, the healthcare worker may select UFUR administration with or without leucovorin. Thus, we aimed to determine the optimal dosing schedule and dosage for the TEGAFIRI regimen in the metastatic colorectal setting. Previous studies indicated that the TEGAFIRI regimen was well-tolerated (11,14) and, by modulating with leucovorin, the TEGAFIRI and TEGAFOX regimens demonstrated comparable efficiency and safety (10,12,13).

The aim of the pilot study was to evaluate the response rates of different regimens of combination chemotherapy employing TEGAFIRI with or without leucovorin for patients with recurrent or metastatic colorectal cancer. To decrease bias, all of the patients in the present study were analyzed by a single health worker. The results indicated that groups II and III exhibited similar response rates and were preferable to the regimen employed in group I. Furthermore, the response rates in groups II and III were similar to those of previous studies, which employed the FOLFIRI regimen with leucovorin (11,13,34). Grade III/IV diarrhea, alopecia and hematologic side effects were acceptable and similar in the three groups. Subsequently, the present study evaluated the response of a larger cohort to 150 mg/m^2^ irinotecan every two weeks with continuous UFUR and leucovorin without interruption, and demonstrated similar side-effect and survival benefits, such that continuous UFUR and leucovorin without interruption appeared to be essential for improved patient survival.

This type of combination chemotherapy is advantageous as it requires no hospital admission, has a shorter injection time, does not require any additional apparatus for injection, is well tolerated by the majority of patients, and exhibits acceptable hematological and non-hematological side effects. However, this regimen is associated with poor patient compliance due to a number of reasons; for example, the irinotecan injection is associated with nausea and vomiting, which may interfere with the desire to self-administer oral agents, as well as vomiting and diarrhea, which may decrease the actual intake of oral agents.

Despite the possibility of the abovementioned disadvantages occurring, the pilot and the present study indicated similar response rates to the TEGAFIRI regimen compared with previous reports in the literature. For example, TEGAFIRI results in satisfactory response rates and patients report tolerable side effects. Furthermore, continuous UFUR administration without interruption appeared to result in improved outcomes compared with the intermittent administration of UFUR, and leucovorin is an essential component of the treatment regimen.

In conclusion, TEGAFIRI combination chemotherapy is a satisfactory alternative therapy to the FOLFIRI regimen, producing acceptable response rates for recurrent or metastatic colorectal cancer patients. The present study recommends that TEGAFIRI should be administered in combination with leucovorin and oral UFUR administration should not be interrupted during treatment.

## Figures and Tables

**Table I:** Response rate and outcome of the three groups from the pilot study. Adapted from the pilot study by Hsu (23).

	Group I (n=11)		Group II (n=11)		Group III (n=11)	
			
Response	n	%	n	%	n	%
Complete response	0	0.0	2	18.2	2	18.2
Partial response	3	27.3	4	36.4	4	36.4
Mortalities	9	81.8	9	81.8	8	72.7
Survival range, months	6–30		5–31		5–33	

**Table II:** Patient demographic data of the present study. The median age of the patients was 61 years (range, 21–81 years).

	Patients (n=113)	
	
Characteristic	n	%
Gender		
Male	56	49.6
Female	57	50.4
Site of the primary tumor		
Colon	49	43.4
Rectum	63	55.8
Two locations	1	0.9
Position of the primary tumor		
Cecum	3	2.7
Ascending colon	12	10.6
Transverse colon	5	4.4
Descending colon	7	6.2
Sigmoid colon	21	18.6
Anus	63	55.8
Two locations	2	1.8
Dukes’ stage of the primary tumor		
B	19	16.8
C	47	41.6
D	47	41.6

**Table III:** Adverse effects experienced by patients in the present study exhibiting an overall toxicity grade of III–IV.

	Patients (n=113)	
	
Adverse effect	n	%
Non-hematological		
Diarrhea	22	19.5
Vomiting	28	24.8
Alopecia	79	69.9
Nausea	45	39.8
Hematological		
Anemia	34	30.1
Neutropenia	67	59.3
Febrile neutropenia	5	4.4
Thrombocytopenia	12	10.6
